# An emergency cash transfer program promotes weight gain and reduces acute malnutrition risk among children 6-24 months old during a food crisis in Niger

**DOI:** 10.7189/jogh.08.010410

**Published:** 2018-06

**Authors:** Jessica Bliss, Kate Golden, Leila Bourahla, Rebecca Stoltzfus, David Pelletier

**Affiliations:** 1 Center for Global Health, Oregon State University, Corvallis, Oregon, USA; 2 Concern Worldwide, Dublin, Ireland; 3 Concern Worldwide, Niamey, Niger; 4 Division of Nutritional Sciences, Cornell University, Ithaca, New York, USA

## Abstract

**Background:**

Assessment of the impact of emergency cash transfer programs on child nutritional status has been difficult to achieve due to the considerable logistic and ethical constraints that characterize humanitarian settings.

**Methods:**

We present the findings from a quasi-experimental longitudinal study of a conditional emergency cash transfer program implemented by Concern Worldwide in 2012 during a food crisis in Tahoua, Niger, in which the use of a concurrent control group permits estimation of the program’s impact on child weight gain. Program beneficiaries received three transfers totaling approximately 65% of Niger’s gross national per capita income; mothers attended mandatory sessions on child and infant feeding and care practices. Dietary and anthropometric data from 211 vulnerable households and children targeted by the intervention were compared with 212 similarly vulnerable control households and children from the same 21 villages. We used multilevel mixed effects regression to estimate changes in weight and weight-for-height Z scores (WHZ) over time, and logistic regression to estimate the probability of acute malnutrition.

**Results:**

We found the intervention to be associated with a 1.27 kg greater overall weight gain (*P* < 0.001) and a 1.82 greater overall gain in WHZ (*P* < 0.001). The odds of having acute malnutrition at the end of the intervention were 25 times higher among children in the comparison group than those in households receiving cash (*P* < 0.001).

**Conclusions:**

We conclude that this emergency cash transfer program promoted child weight gain and reduced the risk of acute malnutrition among children in the context of a food crisis. We suspect that the use of strategic conditional terms and a valuable transfer size were key features in achieving this result. Limitations in study design prevent us from attributing impact to particular aspects of the program, and preclude a precise estimation of impact. Future studies of this nature would benefit from pre-baseline measurements, more exhaustive data collection on household characteristics and transfer use, and further investigation into the use of conditional terms in emergency settings.

Acute malnutrition—the rapid loss of muscle and fat tissue due to inadequate dietary intake and/or illness—increases mortality risk for children three to 9-fold, and is responsible for approximately 12.6% of all child deaths [1]. Identified by low mid-upper arm circumference (MUAC), low weight-for-height Z scores (WHZ), and/or the presence of bilateral pitting edema, the condition is most prevalent in crisis-affected communities, where disrupted access to food, water, shelter, and basic services leaves children vulnerable to poor diets and poor health. The prevention and treatment of moderate and severe acute malnutrition (MAM and SAM, respectively) is a focus of humanitarian interventions, which have historically responded to nutrition emergencies through a combination of household food rations, targeted or blanket supplementary feeding programs, and therapeutic feeding programs [2].

The distribution of cash, on its own or in conjunction with other interventions, is another widely used crisis intervention tool. Emergency cash transfer programs (CTPs) are intended to provide immediate relief to crisis-affected households and communities by helping them acquire the goods and services necessary to sustain themselves until the crisis is resolved [3]. They often include the objective of preventing acute malnutrition, although whether they are an appropriate tool for this purpose is debated [4]. Critical evaluation of emergency CTPs is made difficult by logistic and ethical constraints inherent to operating in crisis settings—in circumstances where the need for services is high, interventions cannot be withheld, and research design is at the mercy of complex and rapid intervention rollout [4,5].

Recent studies from Niger highlight potential synergies between emergency CTPs and supplementary food distribution, as well as outstanding questions about whether or how cash alone could influence acute malnutrition risk. Langendorf et al. (2014) describe a quasi-randomized study of seven intervention strategies in which cash, supplementary food, and family food rations were distributed in different combinations to households during an annual hunger gap in Niger [6]. They found that acute malnutrition risk was lowest in households receiving a combination of cash and supplementary foods. Households that received cash alone (no supplementary food or family food ration) had 2-fold higher incidence of acute malnutrition than those that received a combination of interventions. In work by Hoddinott et al. (2014), communities in Niger were randomly allocated to receive cash, food baskets, and food vouchers of equal value to evaluate the modalities’ potential impact on diet and food security [7]. Cash transfer recipient households had less diverse diets and lower food security indicators than those receiving food baskets. In contrast, Fenn et al. (2014) in their cohort study of a cash-only emergency CTP in Niger found that child WHZ, MUAC, dietary diversity, and household indicators of wealth and food security improved during the course of the program [8]. However, due to the pre-post study design and the absence of a control group, the changes cannot be attributed to the CTP. Additional investigation showed that household spending on child food was not associated with child acute malnutrition risk, a finding that challenged the fallible but persistent assumption that food purchase mitigates risk. Rather, risk was highest among children with poor health and those who entered the period of seasonal famine with low WHZ [9].

The study presented here is the result of a unique opportunity seized by a humanitarian organization, Concern Worldwide (hereafter Concern), to evaluate the impact of a conditional emergency CTP on child weight gain and acute malnutrition risk during the 2012 Niger food crisis. This is among the first studies of an emergency CTP to utilize a concurrent control group. The use of a high-value transfer and nutrition-focused conditional terms further distinguish the program from its counterparts. Our objective was to ascertain changes in weight, WHZ, and the probability of acute malnutrition among study participants.

## METHODS

### Context and program design

The national Government of Niger declared an impending food crisis in May 2012, based on drought and famine early warning system projections of poor crop and fodder production due to insufficient rainfall. High food prices were seen as a key contributing factor to the crisis [10]. Similar circumstances in Niger in 2005-2006 and 2008-2009 had proven devastating, with several million people believed to be affected by crop failure, the inability to afford market-sourced foods, and severe food insecurity [11]. These events led to international calls for enhanced governmental and humanitarian agency response to threats of famine in the country and region [12].

In response to the crisis announced in 2012, Concern implemented a conditional emergency CTP in Niger’s Tahoua region to promote child weight gain and prevent acute malnutrition during the projected period of food crisis. The program targeted 81 villages that had exhibited chronic vulnerability to food insecurity between 2005 and 2011, as determined by assessments of fodder and food production, asset ownership, and previous exposure to drought, pestilence, and human and animal epidemics. The Household Economy Approach (HEA) was used to assign each household in the 81 targeted villages to one of four socio-economic categories, based on household size, land and livestock ownership, and household assets [13]. All of the households in the lowest wealth category were initially enrolled in the intervention, totaling 5286 households.

Surplus funding for the program allowed enrollment of 1211 additional households, which were purposively selected from the second-lowest wealth category. Preference was given to those households headed by females, those with a household member with a disability, those with low income, those with few members able to work, and those with non-permanent dwelling types. Not all existing households in the second-lowest wealth ranking were enrolled in the program. The emergency CTP enrolled a total of 6497 households.

Each household enrolled in the emergency CTP received a total of 125 000 CFA (approximately 250 USD) split into three transfers between July and September 2012. The transfers were given to female heads of household to increase the likelihood that the money would be spent to benefit child health and nutrition [14]. The total value of the transfers was equivalent to 65% of the gross national income per capita in Niger in 2012 [15].

Mothers were required to attend a health and nutrition education session prior to each cash transfer distribution in order to receive their household’s transfer; thus the CTP was a conditional program. Session topics included age-appropriate infant and child feeding, the importance of colostrum and breastfeeding, hand washing, and the use and mixing of oral rehydration salts. Cooking demonstrations showed participants how to prepare and integrate protein-rich foods and vegetable purees into meals for children.

### Concurrent nutrition programming

Two nutrition enhancement programs were simultaneously implemented in the Tahoua region, independently of the CTP. First, Community-based Management of Acute malnutrition (CMAM), which provides therapeutic and supplementary feeding to qualifying children, is routinely offered by the Niger Ministry of Health and various humanitarian agencies at the 17 major health facilities in the CTP area. CMAM care includes therapeutic feeding for severe acute malnutrition and targeted supplementary feeding for moderate acute malnutrition. Second, a separate Concern program distributed seeds and fertilisers to the same 81 villages selected for the CTP one month prior to the first cash distribution (June 2012). Village committees distributed 10 kg bundles of millet seeds and fertiliser to individual households that were considered vulnerable and had access to land. The extent to which these households overlapped with those targeted by the CTP is not known.

### Study design

We describe a quasi-experimental non-randomized study with an intervention and a comparison group, designed to estimate the impact of the emergency CTP on the nutritional status of children aged 6-24 months. Our primary outcomes of interest were weight gain, weight gain velocity (g/kg/d) and the presence of acute malnutrition (WHZ<-2 or MUAC<125mm and/or the presence of bilateral pitting edema) according to the 2006 World Health Organization Child Growth Standards [16].

The target sample size for this study, 213 individuals per group, was calculated to detect a difference of 0.5 g/kg/d in weight gain velocity between the intervention groups, adjusting for similarities within villages using a design effect factor of 1.5 as suggested by the Standardized Monitoring and Assessment of Relief and Transitions (SMART) guidelines [17].

### Selection and description of study participants

Of the 81 villages reached by the program, 21 were purposively selected by Concern as study sites. Study staff visited all beneficiary households within the 21 selected villages, and each household that met the eligibility requirements described below was recruited for study participation.

Households were eligible for the study and assigned to the cash intervention group if they (1) were enrolled in Concern’s emergency CTP, (2) occupied the second-lowest wealth category according to the HEA, and (3) had a child aged 6-23 months of age who was not wasted (MUAC>125mm) and had no current symptoms of disease (diarrhea, fever, or cough). To further clarify, eligibility for the study required that the household be one of the 1211 enrolled with surplus funding (see Program Design, above).

A comparison group naturally emerged, as many households in the same 21 villages had comparable wealth status to those selected for the cash intervention group but had not been enrolled in the emergency CTP (see Program Design, above). Households were eligible for the comparison group if they (1) occupied the same wealth category as cash group households (second lowest using the HEA scale), (2) were not enrolled in the emergency CTP, and (3) had a child aged 6-23 months of age without wasting (MUAC>125mm) and had no current symptoms of disease (diarrhea, fever, or cough). Note that while these households occupied the same wealth category as intervention households, their food security status was regarded as less vulnerable and they had not been selected as CTP beneficiaries (see Program Design, above).

MUAC and/or the presence of bilateral pitting edema were used to assess wasting or acute malnutrition status at the time of enrollment.

### Technical information

#### Data collection

Data were collected detailing child and household characteristics by six teams of two trained Concern enumerators at the homes of study participants at three times: once shortly after the start of the intervention and subsequently in approximately one-month increments (hereafter referred to as baseline, midline, and endline surveys). Surveys were written in French and administered in the Hausa language.

Anthropometric measurements were made using standard techniques according to the SMART guidelines [17]. Weight was recorded to the nearest 0.01 kg using an electronic scale. Length of children <87 cm long was measured to the nearest 0.1cm using a baby mat, and height of children ≥87 cm tall was measured using a stadiometer. MUAC tapes were used to measure mid-upper arm circumference to the nearest 0.1mm. Age was determined by birth certificate, if available, or mother’s estimation. Any child who was found to be acutely malnourished (MUAC<125 mm) during the study was referred to the nearest treatment site.

The number of meals and snacks consumed by the target child within the last 24 hours constituted meal frequency. Consumption (yes/no) of any of the seven WHO infant and young child food groups within the last 24h was recorded (grains, legumes, fruits or vegetables rich in vitamin A, eggs, animal flesh foods, dairy, other fruits and vegetables) [18]. Children who had breastfed within the last 24h were recorded as breastfeeding. Symptoms of child illness (diarrhea, cough, difficulty breathing, or fever in the last two weeks, as observed by the mother) were recorded. Current enrollment in therapeutic or supplementary feeding programs was noted. Vaccination history (measles, Penta 3 for diphtheria, tetanus, pertussis, hepatitis B, and polio), and vitamin A supplementation were determined by reviewing the child’s health card. If no card was available, enumerators asked mothers if the child had been vaccinated or supplemented. Mothers provided information about ethnicity, marital status, and the number of cattle, goats, sheep, poultry, camels and hectares of land owned by the household.

#### Informed consent and ethical approval

Verbal and written informed consent was obtained at each survey for each participant. This study was found to be exempt from ethical review by Cornell University’s Internal Review Board (protocol ID# 1302003601), as Concern collected all of the data for this study as part of their routine program monitoring and evaluation activities.

#### Data management and statistics

Data were assembled in Microsoft Excel and analyzed in STATA [19]. WHZ was calculated using the WHO macro for STATA [20].

#### Data screening

We used two methods to screen our anthropometric data. Both methods yield the same conclusions in statistical analysis, with only minor differences in numerical values of model coefficients. Using an inclusive approach, we included data for all study participants whose WHZ measurements were <6 and>-6 WHZ; these are the data presented and analyzed in the main text. We also used a more conservative approach to address any potential measurement issues common to emergency settings [21]. Using the conservative approach, we included data for participants whose changes in linear growth were within a threshold of ±2.5cm plus the maximum plausible linear growth of a child aged 6-24 months over a two-month period according to the WHO standards (5cm)[9,22]. The full results using the conservative approach are presented in **Online Supplementary Document(Online Supplementary Document)**; the results and interpretation do not vary from those presented in the main text.

#### Analysis

We present descriptive statistics (means, standard deviations, frequencies and percentages) of anthropometric and dietary indicators at baseline, midline, and endline. Indicators include age, age category, sex, weight (kg), WHZ, MUAC (mm), breastfeeding status, meal frequency, diet diversity, CMAM enrollment, and health status. We used Student’s *t* test to compare means of continuous variables and Wald Chi-Squared tests to compare frequencies of categorical variables, and present the unadjusted Difference in Differences (DID) for each comparison between the first study interval (baseline to midline), the second interval (midline to endline) and the full interval (baseline to endline). Weight gain velocity (grams gained per kilogram of body weight per day) was calculated for each age category at each interval (baseline to midline, midline to endline, and baseline to endline). We considered *P-*values <0.05 to be statistically significant.

We used two multilevel mixed-effects linear regression models to estimate the effect of the cash transfer program on weight and WHZ, respectively, treating individuals as random effects to account for random variation in individual growth. An interaction term between study arm and time permitted us to estimate effects for each time increment. Model covariates included child sex (male or female, 0 or 1), age (continuous), breastfeeding within last 24 hours (0 or 1), illness in last two weeks (0 or 1), baseline WHZ, Penta vaccination history (0 or 1), and current enrollment in a CMAM feeding program (0 or 1). We used a mixed effects logistic regression model to estimate the odds of developing acute malnutrition (WHZ<-2, MUAC<125mm, or the presence of bilateral pitting edema), using the same interaction term and covariates described above.

## RESULTS

Data from 426 households were collected for this study, 212 in the cash group and 214 in the comparison group, each group representing the same 21 villages. Two households in the comparison group were removed from analysis due to a protocol error in which they mistakenly received transfers at the third cash distribution, and one cash household was excluded during data screening. Program records show that 98% of the cash group attended all of the conditional educational sessions, and that all cash group households received the full amount of the cash transfers.

**Figure 1:**
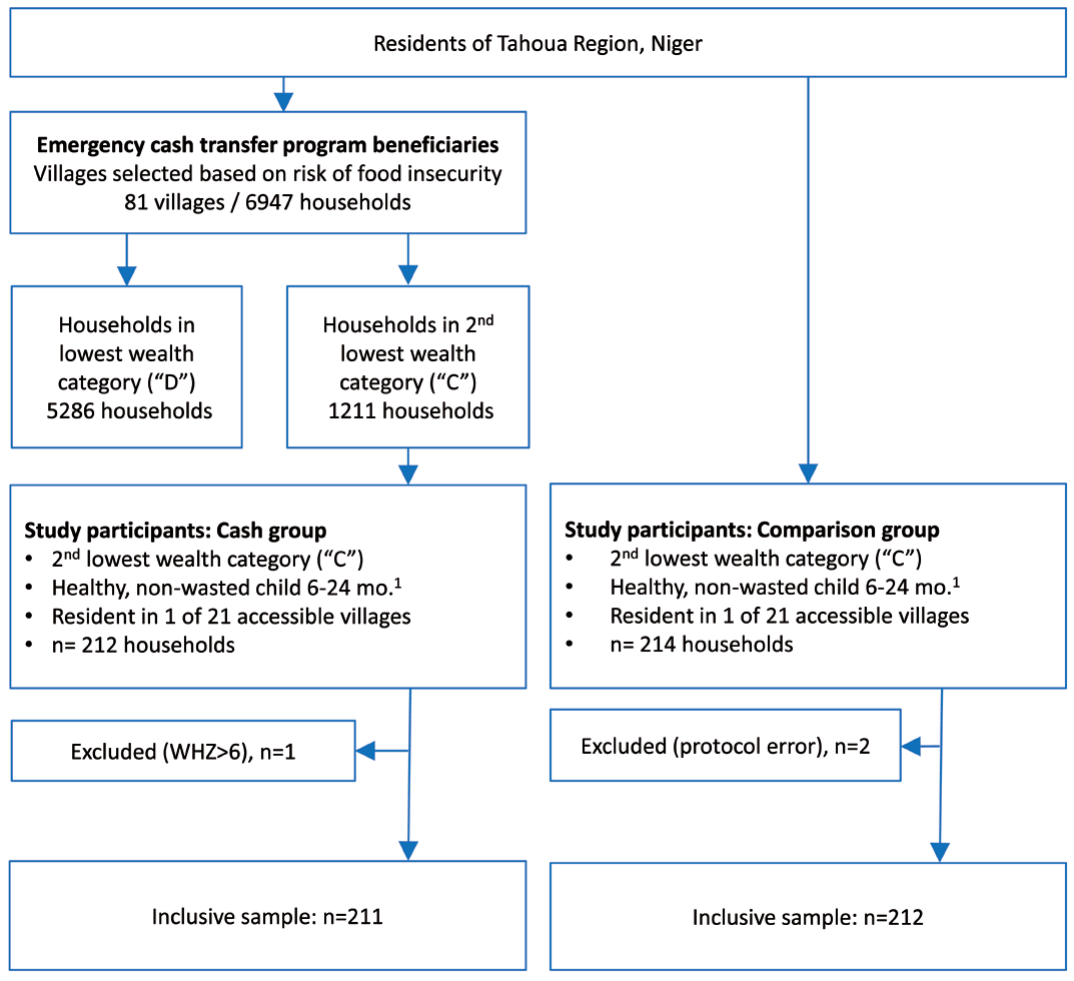
Participants’ flow diagram.

Participant flow is depicted in **Figure 1**.

Over ninety percent of households in each study group were of Hausa ethnicity. The predominant marital status was married monogamous (75% and 65%, cash and comparison respectively), followed by polygamous (23% and 30%), and widowed (3% and 5%). Median land ownership among cash and comparison households was 14 and 11 hectares, respectively, with 25 and 21 percent of households reported owning any livestock at baseline.

Although children in the cash group were lighter, had lower WHZ scores and lower MUAC measurements than comparison group children at baseline, they overcame the relative deficit and exceeded comparison group children on all three indicators by endline (**Table 1**). Weight gain occurred predominantly during the first interval (baseline to midline) and among the oldest children (those 12-24 months old at baseline). Cash group children gained 1.35kg and increased 1.83 WHZ more on average than comparison group children (**Table 2**). Weight gain velocity averaged 3.8 g/kg/d in the cash group and 1.3 g/kg/d in the comparison group (*P* < 0.001); it was highest among cash group children aged 12-24 months during the first interval (6.6g/kg/d) (**Table 3**). No cases of edema were detected at any time during the study.

**Table 1:** Anthropometric and dietary indicators of children in households receiving cash transfers (n = 211) and those in comparison households (n = 212) at baseline, midline, and endline*

Variable	Baseline measure			Midline measure			Endline measure		
	**Cash**	**Comp**	***P*-value†**	**Cash**	**Comp**	***P*-value**	**Cash**	**Comp**	***P*-value**
Age (months)‡	13.7 ± 5.2	13.3 ± 4.9	0.48	15.0 ± 5.2	14.6 ± 4.9	0.44	15.8 ± 5.2	15.4 ± 4.9	0.45
Age category (months):									
6-8	48 (23)	43 (20)		32 (15)	29 (13)		15 (7)	17 (8)	
9-12	42 (20)	42 (20)		39 (18)	34 (16)		44 (21)	34 (16)	
13-24	122 (57)	126 (60)	0.85	128 (60)	141 (67)		135 (64)	149 (71)	
24+s	–	–	–	13 (6)	7 (3)	0.41	19 (8)	11 (5)	0.29
Sex (female)	109 (51)	98 (47)	0.31						
Weight (kg)	7.89 ± 1.0	8.24 ± 1.0	<0.01	9.46 ± 1.5	8.77 ± 1.3	<0.001	9.66 ± 1.5	8.70 ± 1.3	<0.001
WHZ^§^	-1.5 ± 1.1	-1.0 ± 1.1	<0.001	0.3 ± 1.0	-0.7 ± 1.0	<0.001	0.3 ± 1.0	-1.2 ± 1.0	<0.001
MUAC (mm)‖	137 ± 8	139 ± 9	<0.01	137 ± 9	138 ± 10	0.14	142 ± 10	137 ± 10	<0.001
Breastfed# (%)	173 (82)	178 (84)	0.59	166 (79)	172 (81)	0.40	163 (77)	169 (80)	0.54
Meal frequency**	4 ± 2	4 ± 2	0.40	5 ± 2	3 ± 2	<0.001	5 ± 2	4 ± 2	<0.001
Diet diversity††	3 ± 2	2 ± 2	0.06	3 ± 2	2 ± 1	<0.001	4 ± 1	3 ± 1	<0.001
CMAM (%)‡‡	0 (0)	0 (0)	–	7 (3)	5 (2)	0.55	18 (9)	19 (9)	0.88
Recently ill (%)§§	99 (47)	91 (43)	0.41	131 (62)	144 (68)	0.21	127 (60)	139 (66)	0.25
Days passed‖‖	–	–	–	39 ± 3	38 ± 3	<0.01	64 ± 2	63 ± 2	0.001

*Values are mean ± standard deviation or n (%) as appropriate.

†P-values are reported for *t* tests comparing means of continuous variables or χ^2^ tests comparing frequencies of categorical variables.

‡Age was determined by looking at birth certificates when available (n = 181) or estimated by mothers.

§Weight-for-height Z score.

‖Mid-upper arm circumference.

#Any breastfeeding in the last 24 hours.

**Number of meals in the last 24 hours, not including breast milk feeding.

††Number of food groups consumed according to the World Health Organization guidelines for infant and child feeding, which considers 7 groups (grains, legumes, fruits and vegetables rich in Vitamin A, eggs, animal flesh foods, dairy, and other fruits or vegetables).

‡‡Child currently enrolled in Community-based Management of Acute Malnutrition (CMAM) and receiving supplementary foods

§§Presence of diarrhea, fever, difficulty breathing, cough, or any other illness in the last 2 weeks, as reported by the mother.

‖‖Days passed between surveys.

**Table 2:** Difference in difference estimations between children in households receiving cash transfers (n = 211) and those in comparison households (n = 212) at three intervals*

Variable	Baseline to Midline		Midline to endline		Baseline to endline	
	DID†	*P*-value‡	DID	*P*-value	DID	*P*-value
Weight (kg)	1.07	<0.001	0.27	0.17	1.35	<0.001
WHZ§	1.42	<0.001	0.41	<0.01	1.83	<0.001
MUAC‖ (mm)	0.96	0.45	6.0	<0.001	7.0	<0.001
Breastfed# (%)	0	0.93	0	0.99	0	0.93
Meal frequency**	1	<0.001	0	0.91	1	<0.001
Diet diversity††	1	<0.001	0	0.75	1	<0.001
CMAM‡‡ (%)	+1	0.55	–1	0.66	0	0.88
Recently ill§§ (%)	-10	0.14	+4	0.95	+9	0.17
Consumption of WHO food groups‖‖ (%):						
Animal protein	+19	<0.01	+6	0.30	+25	<0.001
Legumes	+22	<0.001	+7	0.28	+27	<0.001
Eggs	+9	0.06	0	0.82	+8	0.09
Cereals	+6	0.10	+1	0.62	+4	0.16
Milk	+9	0.18	-4	0.57	+5	0.44
Vitamin A	+7	0.30	+1	0.89	+8	0.24
Other vegetables	-2	0.37	-1	0.50	-3	0.20

DID – difference in differences

*Values are mean ± standard deviation or n (%) as appropriate.

†Difference in differences (double difference).

‡P-values are reported for difference in difference estimations.

§Weight-for-height Z score.

‖Mid-upper arm circumference.

#Any breastfeeding in the last 24 hours.

**Number of meals in the last 24 hours, not including breast milk feeding.

††Number of food groups consumed according to the World Health Organization guidelines for infant and child feeding, which considers 7 groups (grains, legumes, fruits and vegetables rich in Vitamin A, eggs, animal flesh foods, dairy, and other fruits or vegetables).

‡‡Child currently enrolled in Community-based Management of Acute Malnutrition (CMAM) and receiving supplementary foods.

§§Presence of diarrhea, fever, difficulty breathing, cough, or any other illness in the last 2 weeks, as reported by the mother.

‖‖Percent of sample reporting consumption of each WHO food group in the previous 24 hours.

**Table 3:** Weight gain velocity of children in households receiving cash transfers (n = 211) and those in comparison households (n = 212) at three intervals*

Variable	Baseline to midline			Midline to endline			Baseline to endline		
	**Cash**	**Comp**	***P*-value†**	**Cash**	**Comp**	***P*-value**	**Cash**	**Comp**	**P-value**
Weight gain velocity‡ (g/kg/d)	5.6 ± 6.4	2.3 ± 6.0	<0.001	1.0 ± 0.8	-0.2 ± 2.8	<0.001	3.8 ± 3.9	1.3 ± 3.6	<0.001
Baseline age category (months):									
6-8	3.9 ± 7	4.2 ± 8	0.78	1.1 ± 0.8	-0.3 ± 2.2	<0.001	2.9 ± 4.1	2.3 ± 4.5	0.26
9-12	4.7 ± 6.2	2.8 ± 6.4	0.01	1.0 ± 1.1	0.1 ± 3.0	0.003	3.4 ± 3.8	1.7 ± 4.0	<0.001
13-24	6.6 ± 6.1	1.5 ± 5.1	<0.001	0.9 ± 0.5	-0.3 ± 2.8	<0.001	4.4 ± 3.7	0.8 ± 3.0	<0.001

*Values are mean ± standard deviation.

†*P*-values are reported for *t* tests comparing mean weight gain velocity for each interval.

‡Grams gained per kilogram of body weight per day, using the body weight at the beginning of each respective interval.

Dietary indicators were similar between the two groups at baseline (**Table 1**). Meal frequency increased by one meal more on average among cash group children relative to the comparison group (*P* < 0.001). Dietary diversity also increased; children in the cash group consumed foods from one more WHO food group than those in the comparison group (*P* < 0.001). For both dietary indicators the changes occurred during the first interval (**Table 2**). Cash group children consumption of animal protein and legumes increased by 25% and 27% more than among comparison group children between baseline and endline, and is presumed to account for the relative increase in dietary diversity among intervention children (*P* < 0.001) (**Table 2**).

Although no study participants were identified as having acute malnutrition at baseline according to MUAC or edema indicators, a proportion of them would have been designated as having moderate acute malnutrition had WHZ criteria been used (WHZ<-2). Thirty-one percent of the cash group (n = 67) and 19 percent of the comparison group (n = 39) had WHZ scores less than -2 at baseline (*P* < 0.01). By endline, only 4% of cash group children had acute malnutrition (MUAC<125 or WHZ<-2) (n = 8), whereas rates in the comparison group remained unchanged (n = 42, 20%) (*P* < 0.001).

**Figure 2:**
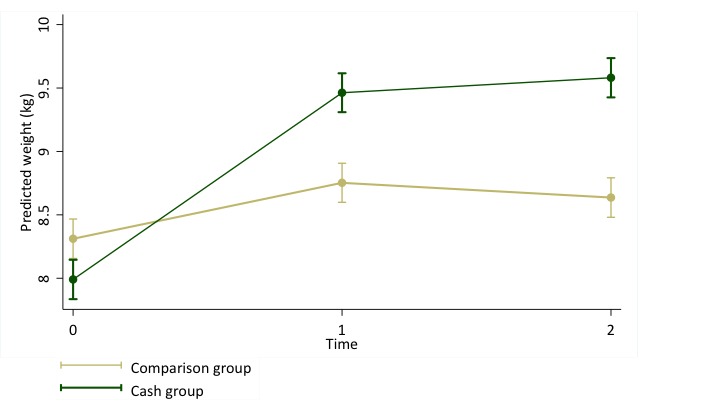
Predicted weight of children in households receiving cash (n = 211) and those in comparison households (n = 212) at baseline, midline and endline. Weight was predicted by a multilevel mixed effects linear regression model, adjusting for child sex (male or female), age (continuous), breastfeeding status, recent illness, baseline weight-for-height Z score, Penta vaccination history, and current enrollment in a therapeutic or supplementary feeding program (n = 1276).

Our mixed effects regression model estimates that the cash intervention was associated with a 1.27kg greater increase in weight during the two months of the intervention, with the majority of weight gain occurring in the first interval (*P* < 0.001). Having a lower WHZ score at baseline, older age, and having had a Penta vaccine were also significantly associated with greater weight gain. Female sex, having recently been breastfed, having recently been ill, and enrollment in a CMAM program were all significantly associated with lower weight gain (**Figure 2**, **Table 4**).

**Table 4:** Associations between cash transfer intervention and child weight, weight-for-height Z scores, and the odds of acute malnutrition

	Weight* (kg)	CI^2^	*P*-value†	WHZ‡	CI	*P*-value	Odds of AM§	CI	*P*-value
**Effect of cash:**									
Baseline-midline	1.03	0.78 to 1.3	<0.001	1.42	1.2 to 1.64	<0.001	0.24	0.11 to 0.59	<0.01
Baseline-endline	1.27	1.01 to 1.53	<0.001	1.82	1.61 to 2.05	<0.001	0.04	0.02 to 0.12	<0.001
Baseline WHZ	0.23	0.16 to 0.31	<0.001	0.40	0.35 to 0.46	<0.001	0.32	0.26 to 0.42	<0.001
Age‖ (months)	0.08	0.07 to 0.10	<0.001	0.01	0.01 to 0.03	0.04	1.00	0.96 to 1.05	0.92
Sex (female)	-0.30	-0.46 to 0.15	<0.001	0.05	-0.07 to 0.17	0.39	0.77	0.51 to 1.18	0.23
Breastfed#	-1.14	-1.31 to 0.98	<0.001	0.10	-0.23 to 0.04	0.14	1.52	0.92 to 2.53	0.10
Recently ill**	-0.14	-0.27 to 0.02	0.02	-0.07	-0.17 to 0.04	0.18	1.22	0.83 to 1.8	0.32
Vaccinated (Penta)††	0.23	0.10 to 0.37	<0.01	-0.02	-0.13 to 0.1	0.76	0.83	0.56 to 1.26	0.38
CMAM‡‡	-0.42	-0.75 to 0.10	0.01	-0.21	-0.48 to 0.05	0.11	3.74	1.56 to 9.03	<0.01

CI – confidence interval, AM – acute malnutrition

*Coefficients produced by a multilevel mixed-effects linear regression model predicting changes in child weight (N = 1267).

†95% confidence interval and P values associated with each model coefficient.

‡Coefficients produced by a multilevel mixed-effects linear regression model predicting changes in child WHZ (weight-for-height Z score) (n = 1267).

§Coefficients are odds ratios produced by a mixed effects logistic regression model predicting the odds of acute malnutrition in children in households receiving cash relative to those in comparison households (n = 1269).

‖Age was determined by looking at birth certificates when available (n = 181) or estimated by mothers.

#Any breastfeeding in the last 24 hours.

**Presence of diarrhea, fever, difficulty breathing, cough, or any other illness in the last 2 weeks, as reported by the mother.

††Child had the Penta 3 vaccination.

‡‡Child currently enrolled in Community-based Management of Acute Malnutrition (CMAM) and receiving supplementary foods.

The intervention was likewise associated with greater WHZ scores and a lower probability of having acute malnutrition. Cash group children experienced a 1.82 greater gain in WHZ than their comparison counterparts, and were 96% less likely to have developed acute malnutrition at endline (**Table 4**). In other words, the odds of having acute malnutrition at endline were 25 times higher among children in the comparison group than in the cash group.

## DISCUSSION

### Key results

This study used a quasi-experimental design to examine the impact of a short-term conditional emergency CTP on child weight gain, diet, and acute malnutrition in the context of a food crisis in Niger. Our findings show that emergency CTPs have the capacity for rapid impact on child ponderal growth—in the program studied here, dietary indicators improved, weight gain accelerated, and the prevalence of acute malnutrition in the cash group declined.

Older children (those 12-24 months at baseline) benefitted the most from the cash intervention in terms of weight gain velocity. This is reminiscent of evidence from non-emergency programs, in which children aged 1-2 years are more responsive than younger children in terms of linear growth [23], and consistent with a recent study that found that low weight gain velocity was more frequent in the first rather than the second year of life among children in a rural setting in the Democratic Republic of Congo [24].

The weight gain velocity that we observed among cash transfer beneficiaries is supported by the parallel improvements in meal frequency and in line with velocities observed among children recovering from acute malnutrition. It falls within the range of weight gain observed for many fortified-blended food interventions used for the treatment of moderate acute malnutrition among children aged 6-24 months, and is comparable to the target weight gain for the average child aged 13-15 months with moderate acute malnutrition (MAM) (5g/kg/d, the equivalent of about 770 kcals/d)[25-28]. And as one should expect, mean weight gain velocities for all but the oldest children were lower than targets for children being treated for severe acute malnutrition (<5g/kg/d are considered poor, 5-10g/kg/d are moderate, >10g/kg/d are good) [29]. While the majority of children in our study were not being treated for acute malnutrition, we find the target weight gains for children with MAM or SAM helpful in confirming the biologic and programmatic plausibility of our study participants.

### Limitations

Households in the cash group were assigned to receive the cash intervention due to their greater vulnerability to food insecurity relative to the households in the comparison group. These assignments were made at baseline by local authorities using indicators described in the methods section, including the number of household members able to work, whether the household was male or female-headed, the presence of disability within the family, marital status, average income, source of income, and type of dwelling. Unfortunately we do not have records of each of the vulnerability indicators used, so we expect that there are unmeasured differences between the treatment groups. If the two groups were in fact similar in terms of vulnerability to food security, they may have had more similar child nutritional and dietary characteristics at baseline, and the differences in growth and acute malnutrition risk between the groups may not have been as stark.

Systematic differences between the treatment groups are not apparent in the available data, with the important exception that children in the intervention arm had poorer nutritional status than those in the comparison group. The inferior nutritional status of intervention children suggests that they may have had greater potential to benefit from a nutritional intervention relative to their peers. While unmeasured systematic differences between treatment groups are undesirable characteristics of this study, we note that the program appropriately targeted more vulnerable households as per their organizational objectives. We propose that given the direction of the difference in weight and WHZ in the treatment groups (with the cash group being more wasted initially less so by endline), the gains in nutritional status in the cash group can be plausibly attributed to the intervention.

We also note that an unknown portion of households in each study group received agricultural materials as part of concurrent programming in the area (see the Methods). Since the harvest season occurred after the CTP concluded, we do not expect that our findings are conflated with any benefits garnered by the agricultural input. An additional limitation of this study is that the first data collection period occurred after the cash transfer intervention had begun. Thus, the actual status of both treatment groups prior to the intervention is unknown, and a precise estimate of the effect of the program on child diet and/or growth is impossible to achieve. Given the first limitation described above, the lagged timing of the initial survey likely produced an underestimate of the effect of the intervention.

### Interpretation

Four convergent lines of evidence support the conclusion that Concern’s conditional emergency CTP had a positive impact on child acute malnutrition. First, weight and WHZ gain in the cash group was significantly greater than that seen in the comparison group, leading to lower odds of acute malnutrition among intervention children by endline. Second, the observed improvements in child ponderal growth are consistent with observed improvements in meal frequency and dietary diversity. These latter feeding behavior changes would’ve been supported by the high value of the transfer (65% of GNI per capita for Niger in 2012) and the high rate of adherence to conditional education sessions on infant and child feeding. Third, the timing of the baseline survey was likely to produce a conservative estimate of the effect size. And fourth, our findings remain stable using both inclusive and conservative means of data screening.

We posit that although Concern’s CTP did not provide supplemental food, high adherence to the nutrition-focused conditional terms may have fulfilled a similar role as food does in other programs and contexts. Because the transfers and the education were delivered in tandem, and no group received just one or the other, the relative importance of the cash vs the conditional terms cannot be separated. However, the high adherence to the conditional terms of the program should not be overlooked as a potentially critical key to the apparent success of this program. Our data do not allow us to test the value of the conditional terms; however evidence from non-emergency CTPs and behavior change interventions supports the hypothesis that the sessions could have led to positive changes in child feeding and care practices [30].

The value of the cash transfer was high relative to the GNI per capita and we suspect this was likely a factor in the program’s success. Fenn et al. examined the relationship between transfer amount and child wasting and found that larger cash transfers had the greatest effect [31]. We know from studies of long-term, non-emergency CTPs that the value of transfers is a determinant of whether beneficiaries adhere to conditions, and conceivably, whether the program has an impact on child nutrition outcomes [32,33]. We suspect that the large value of the transfers in our study may have influenced beneficiaries’ ability and motivation to make impactful changes to their food purchasing and provisioning behaviors.

### Generalizability

We are not aware of other conditional programs implemented in emergency settings, but expect that under similar circumstances—namely similarities in target populations, market conditions, and CTP structure—the success of Concern’s program could be both replicated and improved upon. Meanwhile, the evidence from unconditional emergency programs is mixed. Work by Langendorf (2014) and by Hoddinott (2014) in Niger suggests that the use of food as aid may have distinct benefits that are not necessarily replicable with cash alone [6,7]. Fenn et al., however, found a positive effect of a cash-alone intervention; they used a four-armed longitudinal cluster randomized trial to test the effects of two unconditional cash transfer amounts, a fresh food voucher, and a control group with no cash-related interventions on child nutritional outcomes in rural Pakistan. Children in households receiving the largest cash amount had significantly lower odds of acute malnutrition at six months than those in the other groups [31]. Had cash been paired with nutrition-specific conditions it is possible that outcomes in previous studies would have had an even greater impact on child nutrition outcomes.

## CONCLUSIONS

In conclusion, we found evidence that a high-value, conditional cash transfer program is capable of promoting child weight gain among vulnerable children in the context of a food crisis. Future programs would benefit from studies with more exhaustive household and expenditure data collection, and deliberate testing of how conditional terms can be reasonably met and maximized in humanitarian settings.
