# Supplementary material for: An emergency cash transfer program promotes weight gain and reduces acute malnutrition risk among children 6-24 months old during a food crisis in Niger
Source: J Glob Health. 2018 Feb 17;8(1):010410. doi: 10.7189/jogh.08.010410 (PMC5825977; doi:10.7189/jogh.08.010410)
Supplement: Online Supplementary Document [file jogh-08-010410-s001.pdf]

Online Supplementary Document

Bliss et al. An emergency cash transfer program promotes weight gain and reduces acute malnutrition risk among children 6-24 months old during a food crisis in Niger

J Glob Health 2018;8:010409

SUPPLEMENTARY TABLE 1 Anthropometric and dietary indicators of children in households receiving cash transfers (n=150) and those in comparison households (n=138) at baseline, midline, and endline[1] (Conservative sample: n=150 cash households, n=138 comparison households)

Variable	Baseline measure			Midline measure			Endline measure		
	Cash	Comp	P[2]	Cash	Comp	P	Cash	Comp	P
Age [3] (mo.)	13.6±5	13.7±5	0.96	14.9±5.0	14.9±4.7	0.97	15.7±5.0	15.8±4.7	
Age category									
6-8 mo.	33 (22)	21 (15)		22 (15)	15 (11)		12 (8)	7 (5)	
9-12 mo.	31 (21)	31 (23)		25 (17)	20 (15)		27 (18)	19 (14)	
13-24 mo.	86 (57)	86 (62)	0.34	95 (63)	97 (70		100 (67)	104 (75)	
Sex (female)	75 (50)	63 (46)	0.46	8 (5)	6 (4)	0.64	11 (7)	8 (6)	0.43
Weight (kg)	7.9±1.0	8.2±1.0	0.02	9.5±1.5	8.8±1.2	<0.01	9.7±1.5	8.7±1.2	<0.01
WHZ [4]	-1.5 ± 1.1	-1.0 ± 1.1	<0.01	0.3±1.0	-0.7±1.0	<0.01	0.3±0.9	-1.2±1.0	<0.01
MUAC [5] (mm)	137 ± 8	139 ±9	0.02	136±9	139±10	0.04	141±9	138±10	<0.01
Breastfed [6] (%)	124 (83)	120 (87)	0.30	120 (80)	111 (80)	0.93	118 (79)	107 (78)	0.84
Meal frequency [7]	4 ± 2	4 ± 2	0.33	4±2	3±2	<0.01	5±2	4±2	<0.01
Diet diversity [8]	3 ± 2	2 ± 2	0.16	3±1	2±1	<0.01	4±1	3±1	<0.01
CMAM [9] (%)	0 (0)	0 (0)	-	6 (4)	1 (1)	0.07	15 (10)	12 (9)	0.69
Recently ill [10] (%)	77 (51)	56 (41)	0.07	91 (60)	90 (65)	0.43	87 (58)	91 (66)	0.17
Days passed [11]	-	-	-	39±4	38±3	<0.01	64±3	63±2	<0.01

[1] Values are mean ± standard deviation or n (%) as appropriate. [2] P-values are reported for t-tests comparing means of continuous variables or Chi-squared tests comparing frequencies of categorical variables. [3] Age was determined by looking at birth certificates when available (n=134) or estimated by mothers. [4] Weight-for-height Z score. [5] Mid-upper arm circumference. [6]Any breastfeeding in the last 24 hours. [7] Number of meals in the last 24 hours, not including breast milk feeding. [8] Number of food groups consumed according to the World Health Organization guidelines for infant and child feeding, which considers 7 groups (grains, legumes, fruits and vegetables rich in Vitamin A, eggs, animal flesh foods, dairy, and other fruits or vegetables). [9] Child currently enrolled in Community-based Management of Acute Malnutrition (CMAM) and receiving supplementary foods. [10] Presence of diarrhea, fever, difficulty breathing, cough, or any other illness in the last 2 weeks, as reported by the mother. [11] Days passed between surveys.

SUPPLEMENTARY TABLE 2. Difference in difference estimations between children in households receiving cash transfers (n=150) and those in comparison households (n=138) at three intervals[1] (Conservative sample: n=150 cash households, n=138 comparison households)

Variable	Baseline to Midline		Midline to Endline		Baseline to Endline	
	DID [2]	P [3]	DID	P	DID	P
Weight (kg)	0.90	<0.001	0.32	0.17	1.22	<0.001
WHZ [4]	1.44	<0.001	0.48	0.003	1.92	<0.001
MUAC [5] (mm)	0.14	0.93	5.6	<0.001	5.7	<0.001
Breastfed [6] (%)	5	0.47	1	.90	6	0.40
Meal frequency [7]	1	0.002	0	0.81	1	0.002
Diet diversity [8]	1	0.001	0	0.63	1	<0.001
CMAM [9] (%)	3	0.06	-2	0.61	1	0.71
Recently ill [10] (%)	-16	0.05	-3.4	0.68	-19	0.02

[1] Values are mean ± standard deviation or n (%) as appropriate. [2] Difference in differences (double difference). [3]P-values are reported for difference in difference estimations. [4] Weight-for-height Z score. [5] Mid-upper arm circumference. [6]Any breastfeeding in the last 24 hours. [7] Number of meals in the last 24 hours, not including breast milk feeding. [8] Number of food groups consumed according to the World Health Organization guidelines for infant and child feeding, which considers 7 groups (grains, legumes, fruits and vegetables rich in Vitamin A, eggs, animal flesh foods, dairy, and other fruits or vegetables). [9]Child currently enrolled in Community-based Management of Acute Malnutrition (CMAM) and receiving supplementary foods. [10] Presence of diarrhea, fever, difficulty breathing, cough, or any other illness in the last 2 weeks, as reported by the mother.

Supplementary Table 3. Weight gain velocity of children in households receiving cash transfers (n=150) and those in comparison households (n=138) at three intervals[1] (Conservative sample: n=150 cash households, n=138 comparison households)

Variable	Baseline to Midline			Midline to Endline			Baseline to Endline		
	Cash	Comp	P [2]	Cash	Comp	P	Cash	Comp	P
Weight gain velocity [3](g/kg/d)	5.3±4.5	2.5±4.3	<0.001	0.9±0.5	-0.5±2.3	<0.001	3.7±2.7	1.3±2.6	<0.001
Age category									
6-8 mo.	2.7±4.6	1.±5	0.28	1.0±0.2	-1.1±1.7	<0.001	2.1±2.9	0.2±3.1	0.03
9-12 mo.	5.4±4.1	3.0±4.7	0.04	0.8±0.6	-0.3±2.0	<0.01	3.7±2.4	1.6±2.7	<0.01
13-24 mo.	6.3±4.2	2.6±3.9	<0.001	0.9±0.6	-0.4±2.5	<0.001	4.2±2.5	1.4±2.4	<0.001

[1] Values are mean ± standard deviation. [2] P-values are reported for t-tests comparing mean weight gain velocity for each interval. [3] Grams gained per kilogram of body weight per day, using the body weight at the beginning of each respective interval.

Supplementary Table 4. Associations between cash transfer intervention and child weight, weight-for-height Z scores, and the odds of acute malnutrition (Conservative sample: n=150 cash households, n=138 comparison households)

	Weight [1]	CI [2]	P [2]	WHZ [3]	CI	P	Odds of AM [4]	CI	P
Effect of cash									
Baseline-Midline	0.92	0.68-1.17	<0.001	1.43	1.17-1.71	<0.001	0.23	0.09-0.67	0.01
Baseline-Endline	1.23	0.99-1.49	<0.001	1.91	1.65-2.19	<0.001	0.05	0.02-0.16	<0.001
Baseline WHZ	0.22	0.15-0.31	<0.001	0.43	0.37-0.5	<0.001	0.34	0.26-0.47	<0.001
Age [5] (mo)	0.13	0.12-0.16	<0.001	0.02	0.01-0.04	0.04	0.98	0.93-1.04	0.38
Sex (f)	-0.32	-0.51--0.15	<0.001	0.06	-0.09-0.2	0.43	0.67	0.41-1.13	0.13
Breastfed [6]	-0.49	-0.68--0.32	<0.001	-0.04	-0.22-0.13	0.61	1.24	0.66-2.36	0.50
Recently ill [7]	-0.08	-0.21-0.05	0.21	-0.07	-0.2-0.05	0.24	1.38	0.87-2.22	0.18
Vaccinated (Penta) [8]	0.05	-0.09-0.2	0.48	-0.01	-0.15-0.13	0.89	0.87	0.52-1.45	0.59
CMAM [9]	-0.24	-0.56-0.09	0.15	-0.09	-0.41-0.23	0.58	4.04	1.41-11.64	0.01

[1] Coefficients produced by a multilevel mixed-effects linear regression model predicting changes in child weight (N=863). [2] 95% confidence interval and P values associated with each model coefficient. [3] Coefficients produced by a multilevel mixed-effects linear regression model predicting changes in child WHZ (weight-for-height Z score) (N=863). [4] Coefficients are odds ratios produced by a mixed effects logistic regression model predicting the odds of acute malnutrition in children in households receiving cash relative to those in comparison households (N=864). [5]Age was determined by looking at birth certificates when available (n=134) or estimated by mothers. [6]Any breastfeeding in the last 24 hours. [7] Presence of diarrhea, fever, difficulty breathing, cough, or any other illness in the last 2 weeks, as reported by the mother. [7] Child had the Penta 3 vaccination. [8]Child currently enrolled in Community-based Management of Acute Malnutrition (CMAM) and receiving supplementary foods.

Supplemental Figure 1- Participant Flow (Conservative sample)

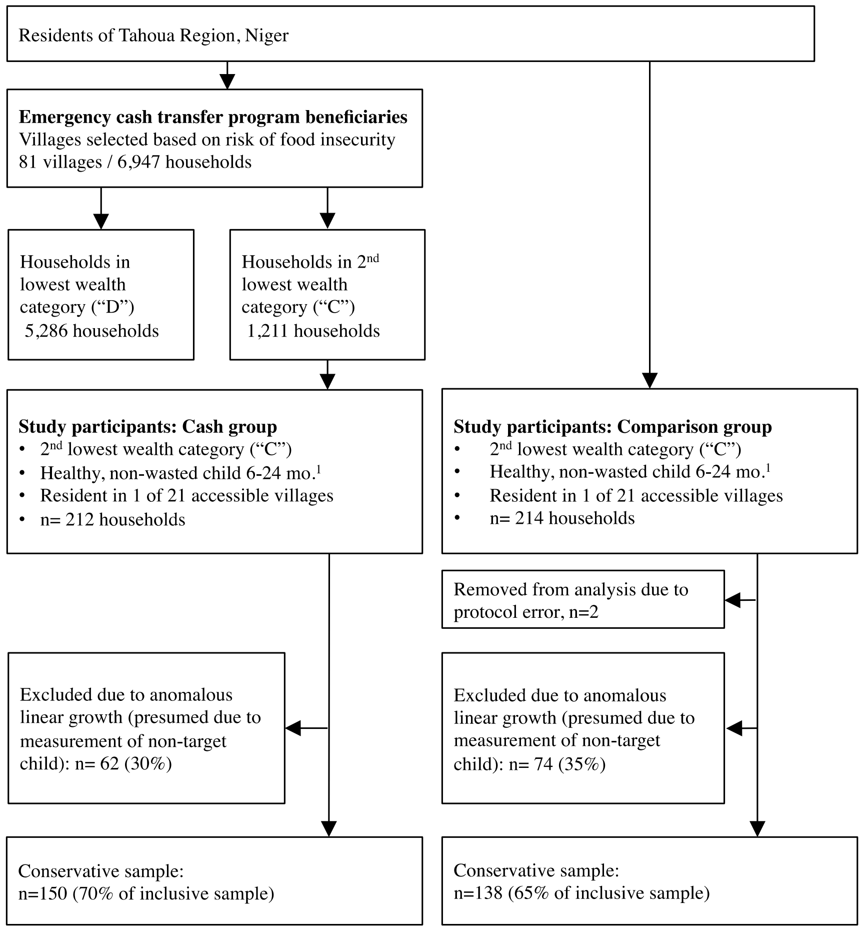

Supplemental Figure 2

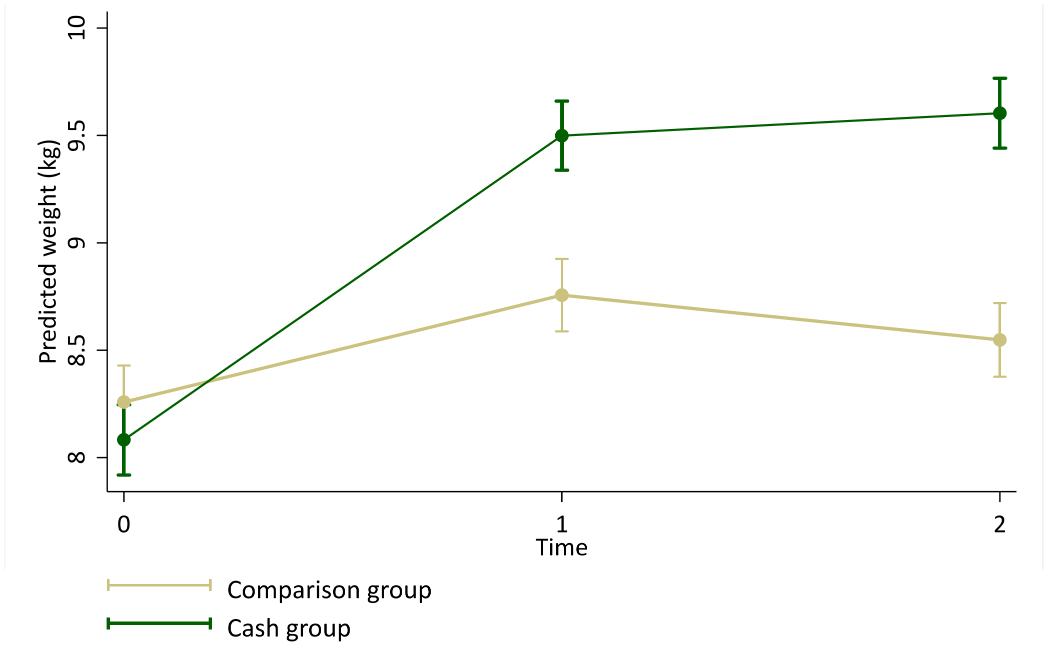

Supplemental Figure 2. Predicted weight of children in households receiving cash (n=150) and those in comparison households (n=138) at baseline, midline, and endline. Weight was predicted by a multilevel mixed effects linear regression model, adjusting for child sex (male or female), age (continuous), breastfeeding status, recent illness, baseline weight-for-height Z score, Penta vaccination history, and current enrollment in a therapeutic or supplementary feeding program (N=863).
